# Synthesis of Isothianaphthene (ITN) and 3,4-Ethylenedioxy-Thiophene (EDOT)-Based Low-Bandgap Liquid Crystalline Conjugated Polymers

**DOI:** 10.3390/ma6062218

**Published:** 2013-05-30

**Authors:** Aohan Wang, Kohsuke Kawabata, Hiromasa Goto

**Affiliations:** Faculty of Pure and Applied Sciences, Division of Materials Science, University of Tsukuba, Tsukuba, Ibaraki 305-8573, Japan; E-Mails: s-awang@ims.tsukuba.ac.jp (A.W.); barbapapa.610@gmail.com (K.K)

**Keywords:** π-conjugated polymers, isothianaphthene, liquid crystals, Migita-Kosugi-Stille coupling, pyrimidine

## Abstract

Copolymers, consisting of isothianaphthene and phenylene derivatives with liquid crystal groups, were synthesized via Migita-Kosugi-Stille polycondensation reaction. IR absorption, UV-vis optical absorption, and PL spectroscopy measurements were carried out. Thermotropic liquid crystallinity of the polymers with bandgap of ~2.5 eV was confirmed.

## 1. Introduction

Liquid crystal (LC) materials have provided interesting studies of molecular aggregation. The aggregation via intermolecular interaction and molecular packing are comparable with crystals; however, LC materials show liquid fluidity. There are many LC compounds with a variety of molecular structures. In general, LC materials consist of a rigid mesogen core and flexible side chains. In the case of polymer LCs, main-chain-type [1] and side-chain-type polymer LC [2] have been studied. Aromatic groups of a monomer repeat unit play the role of mesogenic core in the case of main-chain type polymer LCs. Side-chain-type LC polymers have side-chain LC groups connected to a main chain via a flexible, long, methylene spacer. Main-chains can be aligned through orientation of the side-chain LC. In the case of side-chain-type polyvinylene LCs, molecular movement of the main-chain and LC side-chains are decoupled due to the long flexible alkyl spacers.

π-Conjugated polymers have been paid attention because of their electro-activities [3]. Some of the π-conjugated polymers can show LC behavior in appropriate conditions. As for aromatic type π-conjugated polymers, the main-chains consist of phenylenes or hetero-rings such as thiophene and pyrrole. The aromatic type conjugated polymers often show main-chain-type liquid crystallinity.

In our previous study, side-chain-type aromatic conjugated polymers have been synthesized [4,5]. Introduction of a mesogen into the main-chain results in a decrease of electron affinity and an increase of ionization potential, resulting in an expansion of the bandgap. On the other hand, employment of low bandgap characteristics can compensate the depression of the π-conjugation derived from the introduction of LC groups. Therefore, low-bandgap LC conjugated polymers can effectively function as an electroactive liquid crystal in the present study. The LC properties can allow polarized emission. In particular, low-bandgap LC polymers have a possibility of polarizing emission in NIR range. We apply isothianaphthene (ITN) or 3,4-ethylenedioxythiophene (EDOT) to the main-chain unit.

**Figure 1:**
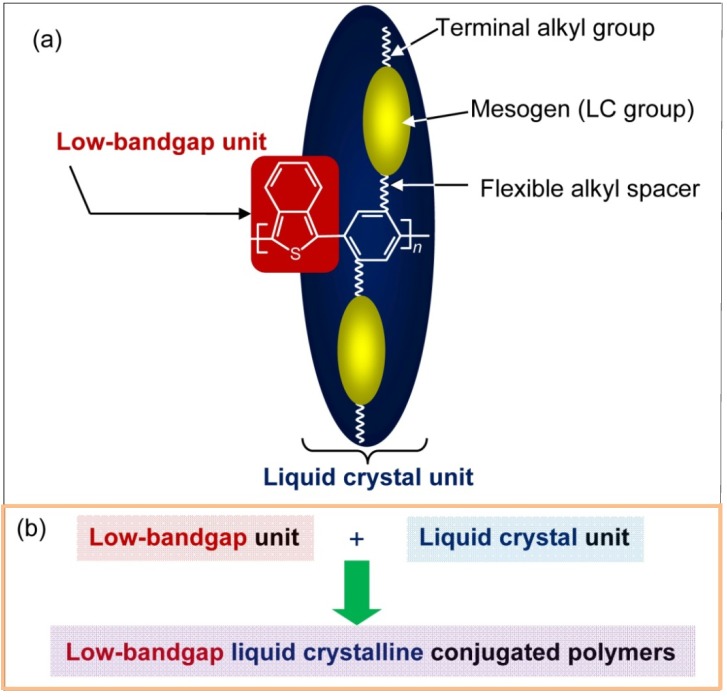
(**a**) Molecular structure of low-bandgap liquid crystalline conjugated polymer; (**b**) A concept of low-bandgap liquid crystalline conjugated polymers.

ITN and EDOT are low-bandgap units for conjugated polymers. The polymer structures synthesized in the present study and a concept of low-bandgap liquid crystalline conjugated polymers are displayed in Figure 1. The polymers consist of a low-bandgap unit and an LC unit. Mesogens were introduced on both sides to strengthen liquid crystallinity. In this communication, we report synthesis of low bandgap liquid crystalline polymers having ITN and EDOT units in the main-chain.

## 2. Experimental Section

### 2.1. Materials and Methods

4-[2-(4-Decyl-3-fluoro-phenyl)-pyrimidin-5-yl]-phenol (Midori Chemical, Japan) was employed for the LC side chain. Compound 3 was prepared by the Mitsunobu reaction. 1,3-Di(trimethylstannyl)isothianaphthene (ITN(SnMe_3_)_2_) [6], and 2,5-di(tributylstannyl)-3,4-ethylenedioxythiophene (EDOT(SnBu_3_)_2_) [7] were prepared by the previously reported method. Note that EDOT(SnBu_3_)_2_ was obtained with quite low yield after purification (6%) because a large part of the desired compound was decomposed through the column chromatography in the purification process. All of the synthetic processes for these compounds should be carried out in argon atmosphere, without exception. Also, purification of both ITN(SnMe_3_)_2_ and EDOT(SnBu_3_)_2_ needs much effort to prevent polymerization. Molecular weights of the polymers were determined by gel permeation chromatography (GPC) with MIXED-D HPLC column (Polymer Laboratories), PU-980 HPLC pump (Jasco) and MD-915 multiwavelength detector (Jasco), with tetrahydrofuran (THF) used as the solvent, with the instruments calibrated by polystyrene standard. Infrared (IR) spectroscopic measurements were carried out with FT/IR-300 spectrometer (Jasco). Ultraviolet-visible (UV-vis) absorption spectra and photoluminescence spectra of the polymers in chloroform were obtained with V-630 UV-vis optical absorption spectrometer (Jasco) and F-4500 fluorescence spectrophotometer (Hitachi). Optical texture observation was performed with ECLIPSE LV100 polarizing optical microscope (POM) (Nikon) equipped with TM-600PM hot stage (Linkam). Differential scanning calorimetry (DSC) measurements were carried out with Seiko EXSTAR7000.

### 2.2. Monomer Synthesis

**Scheme 1:**
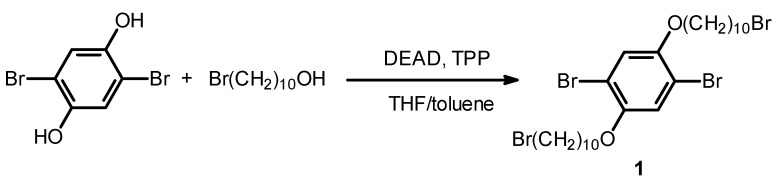
Synthesis of Compound 1. DEAD = diethyl azodicarboxylate, TPP = triphenylphosphine, THF = tetrahydrofuran.

Compound 1 was synthesized with the Mitsunobu reaction, as shown in Scheme 1. Triphenylphosphine (2 g, 7.6 mmol) was added to a solution of diethyl azodicarboxylate (DEAD, 2.2 mol/L in toluene solution 6.8 mL) in 5 mL of THF. The mixture was stirred for 30 min while the color of the solution turned dark red. Then, 2,5-dibromobenzene-1,4-diol (2 g, 7.5 mmol), bromodecanol (3.5 g, 14.8 mmol) were added to the solution, and stirred for 12 h at room temperature. After evaporation of the solvent, the mixture was washed with water thoroughly, and extracted with dichloromethane. The crude product was purified with column chromatography (silica gel, dichloromethane) to afford 1.12 g of white powder (Y = 15.4%). Compound 3 was synthesized with the Mitsunobu reaction, according to the previously reported method [8].

**Scheme 2:**
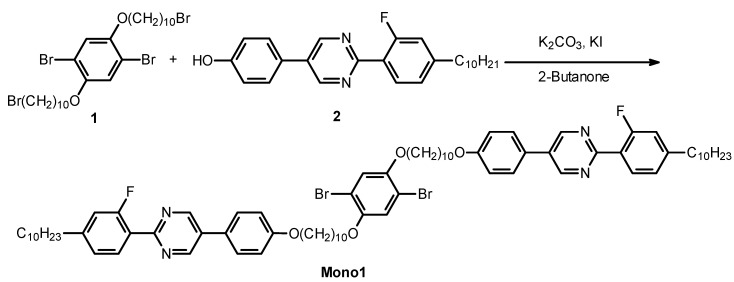
Synthesis of mono1.

Mono1 was synthesized with the Williamson etherification, as shown in Scheme 2. A mixture of Compound 1 (0.2 g, 0.28 mmol), 4-[2-(4-decyl-2-fluoro-phenyl)-pyrimidin-5-yl]-phenol (2) (0.23 g, 0.566 mmol), K_2_CO_3_ (1 g, 7.2 mmol), and KI (1.2 g, 7.2 mol) in 2-butanone (2 mL) was stirred for 24 h at 60 °C. The solvent of the reaction mixture was evaporated. The solid mixture was dissolved in dichloromethane, washed with water thoroughly, and extracted with dichloromethane. After evaporation, the crude product was purified with column chromatography (silica gel, dichloromethane) to afford 0.1 g of white powder (Y = 27%).

**Scheme 3:**
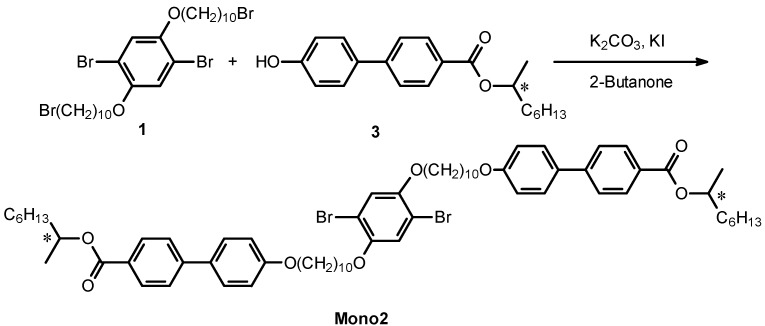
Synthesis of mono2. * = stereogenic center with (*S*)-configuration.

**Figure 2:**
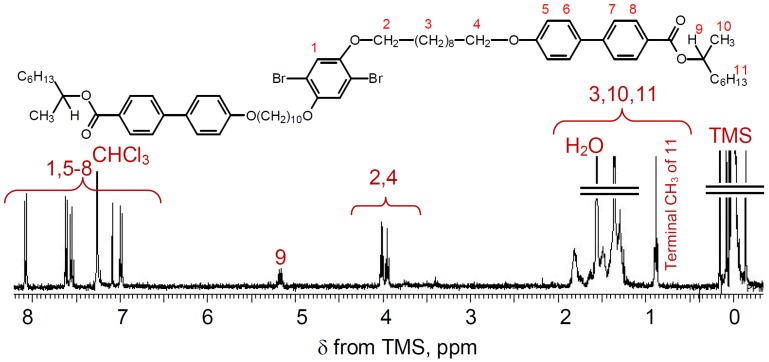
^1^H NMR spectrum of mono2 in CDCl_3_. TMS = tetramethylsilane.

Mono2 was synthesized with the Williamson etherification, as shown in Scheme 3. A mixture of compound 1 (1.25 g, 1.7 mmol), 4′-hydroxy-biphenyl-4-carboxylic acid 1-methyl-heptyl ester with (*S*)-configuration at the stereogenic center (3) (1.58 g, 4.8 mmol), K_2_CO_3_ (0.49 g, 3.5 mmol), and KI (60 mg, 0.36 mmol) in 2-butanone (30 mL) was stirred for 24 h at 80 °C. The solvent was evaporated. The mixture was dissolved in dichloromethane and washed with water thoroughly, and extracted with dichloromethane. After evaporation, the crude product was purified with column chromatography (silica gel, dichloromethane) to afford 0.51 g of white powder (Y = 25%). ^1^H NMR of mono2 in CDCl_3_ is shown in Figure 2. A proton at the stereogenic center is observable at 5.35 ppm from tetramethylsilane (TMS) as an internal standard. Methylene protons adjacent to the oxygen atom are detected at around 4 ppm. Aromatic protons appear at the low magnetic region.

### 2.3. Polymerization

**Scheme 4:**
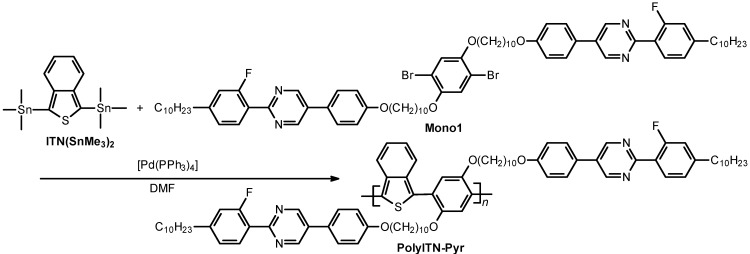
Synthesis of polyITN-Pyr. DMF = *N*,*N*-dimethylformamide.

**Scheme 5:**
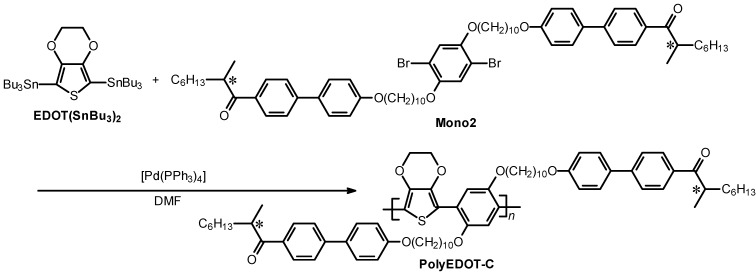
Synthesis of polyEDOT-C. * = stereogenic center with (*S*)-configuration, DMF = *N*,*N*-dimethylformamide. Bu = *n*-butylene moiety.

Notes: **PolyITN-Pyr**, quantity used: mono1 (0.05 g, 0.363 mmol), ITN(SnMe_3_)_2_ (10 mg, 0.363 mmol), DMF (0.4 mL), [Pd(PPh_3_)_4_] (1.6 mg). Y = 51 mg, dark brown power; **PolyEDOT-C**, quantity used: mono2 (69.5 mg, 0.067 mmol), EDOT(BuMe_3_)_2_ (31 mg, 0.067 mmol), DMF (0.3 mL), [Pd(PPh_3_)_4_] (8 mg). Y = 36.1 mg, dark red powder.

The conjugated polymers having LC substituents were prepared via the Migita-Kosugi-Stille polycondensation reaction between ITN(SnMe_3_)_2_ and mono1 (or EDOT(SnBu_3_)_2_ and mono2) in the presence of a Pd(0) complex catalyst (Scheme 4 and Scheme 5). Firstly, equimolar amounts of ITN(SnMe_3_)_2_ and mono1 (or EDOT(SnBu_3_)_2_ and mono2) were mixed into *N*,*N*-dimethylformamide (DMF) under N_2_ flow and refluxed for 30 min. Next, tetrakis(triphenylphosphine)palladium(0) [Pd(PPh_3_)_4_] was added to the mixture and the reaction was allowed to proceed for 2 days at 90 °C. The reaction mixture was then dissolved in a minimal amount of chloroform. The solution was washed with methanol to remove the catalyst and low molecular weight fractions. The red polymer was collected by suction filtration and dried in vacuum to afford the desired product. The polymers thus obtained are abbreviated as polyITN-Pyr (Pyr = pyrimidine) and polyEDOT-C (C = chiral LC).

The GPC results for the polymers are summarized in Table 1. Number-average-molecular weights (*M*_n_) and weight-average-molecular weights (*M*_w_) of polyITN-Pyr were found to be 5150 and 6500 (g/mol), respectively. Dispersity (*M*_w_/*M*_n_) was 1.3. *M*_n_ and *M*_w_ of polyEDOT-C were found to be 5060 and 6160 (g/mol), respectively.

**Table 1:** Polymerization results.

Polymer	*M*_n_ (g/mol) ^a^	*M*_w_ (g/mol) ^a^	*M*_w_/*M*_n_
PolyITN-Pyr	5150	6500	1.3
PolyEDOT-C	5060	6160	1.1

^a^ Relative to polystyrene standard.

## 3. Results and Discussion

### 3.1. IR

**Figure 3:**
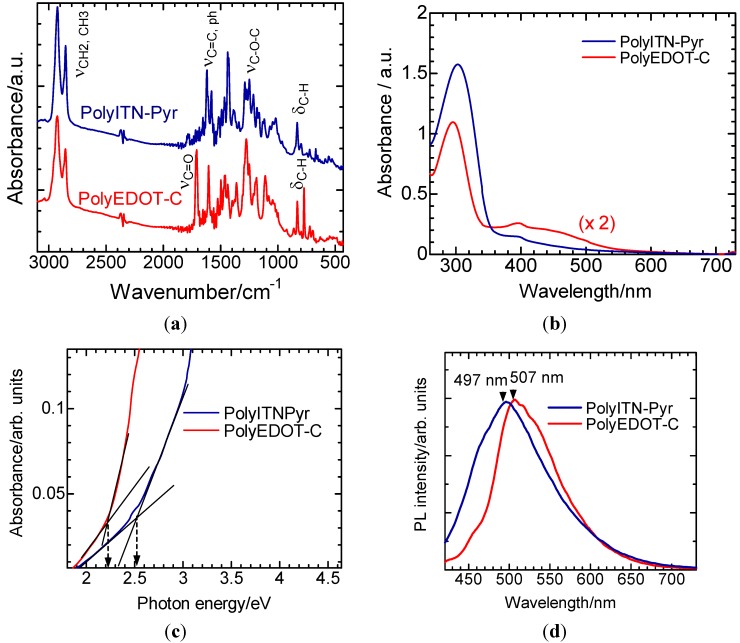
(**a**) FT-IR spectra of the polymers; (**b**) UV-vis optical absorption spectra of the polymers; (**c**) Absorbance as a function of photon energy; (**d**) Photoluminescence spectra.

Figure 3a shows Fourier transform infrared (FT-IR) absorption spectra for the two polymers. The polymers exhibit absorption bands assignable to the flexible alkyl spacer attached to the phenylene and the methyl group (CH_3_ and CH_2_ stretching at *ca*. 2900 cm^−1^). PolyITN-C shows the C=O stretching band at 1711 cm^−1^ due to ester groups in the side chain. An absorption band due to C–O–C stretching is observed at 1276 cm^−1^ for both polymers. Ar–H out-of-plane vibration was observed at 832 cm^−1^. Also, polyITN-C displays Ar–H out-of-plane vibration at 777 cm^−1^.

### 3.2. Ultraviolet-Visible Absorption Spectra

Figure 3b shows the UV-vis optical absorption spectra of the polymers in chloroform solution. PolyITN-Pyr and polyEDOT-C exhibit absorption bands at 302 nm and 293 nm, respectively, due to π–π* transition of the aromatic group of the monomer repeat unit and side-chains. Absorption due to π–π* transition of the main-chain of the polymers is observable at >400 nm. Figure 3c displays optical absorption of the polymers as a function of photon energy. The band-edge bandgaps evaluated from onset of the absorption of polyITN-Pyr and polyEDOT-C are found to be 2.51 eV and 2.21 eV, respectively.

### 3.3. Photoluminescence

Figure 3d shows the photoluminescence (PL) spectra for the two polymers in chloroform solution. The polymers exhibit PL maximum at 507 nm (polyITN-Pyr) and 489 nm (polyEDOT-C).

### 3.4. Polarizing Optical Microscopy Observation

**Figure 4:**
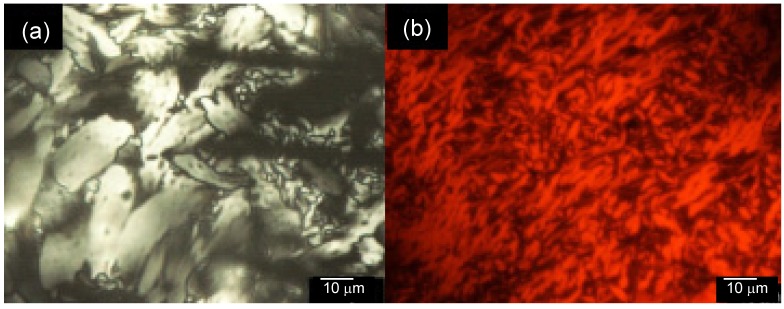
Polarizing optical microscopy (POM) images of the polymers at room temperature. The samples were first melted at 85 °C and gradually cooled to room temperature. (**a**) PolyITN-Pyr; (**b**) polyEDOT-C.

**Figure 5:**
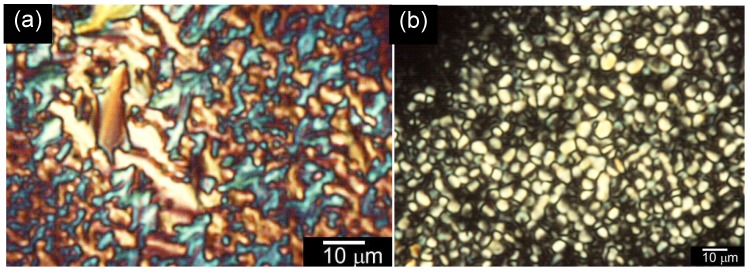
Polarizing optical microscopy (POM) images of PolyITN-Pyr at room temperature. The samples were first melted at 85 °C and gradually cooled to room temperature. (**a**) Insertion of a gypsum first-order red plate; (**b**) Structure resembling small beans.

Optical textures of the polymers were observed with the POM. PolyITN-Pyr and polyEDOT-C show mesophase, as shown in Figure 4. The polyITN-Pyr and the polyEDOT-C show mosaic texture and dark red fan-shaped texture, respectively. In the case of the polyEDOT-C, the side-chains are chiral smectic C (SmC*)-type mesogen. However, the polyEDOT-C shows no tilted LC phase because the main-chain can arrange the side-chains. Insertion of a gypsum first-order red plate under cross Nicol condition demonstrates the colored domain texture of polyITN-Pyr (Figure 5a). The same sample at another part of polyITN-Pyr displays a texture resembling small beans (Figure 5b).

The red color of polyEDOT-C (thick sample between sandwiched glass cell) is derived from π–π* transition of the main-chain. The optical retardation from birefringence was covered by the dark red color. On the other hand, polyITN-Pyr in the present observation was carried out for a thin sample. The sample was sandwiched between two glass plates.

**Figure 6:**
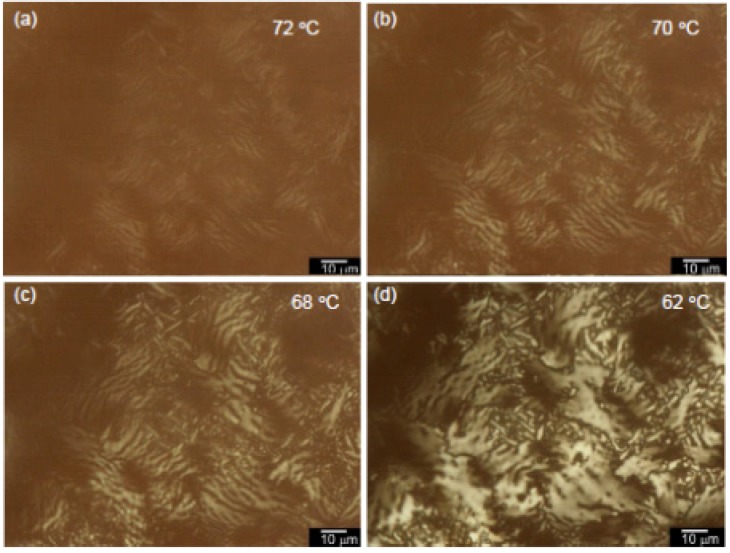
Polarizing optical microscopy (POM) images of polyITN-Pyr from 72 °C to 62 °C in the cooling process.

Figure 6 displays the POM images of polyITN-Pyr from 72 °C to 62 °C in the cooling process. The change in the optical texture of polyITN-Pyr indicates that the polymer first forms fine string-like domains (a), then grow on cooling (b). Next, the domains are unified to large fan-shaped domains, and the large fan-shaped domains grow into a mosaic shape.

**Figure 7:**
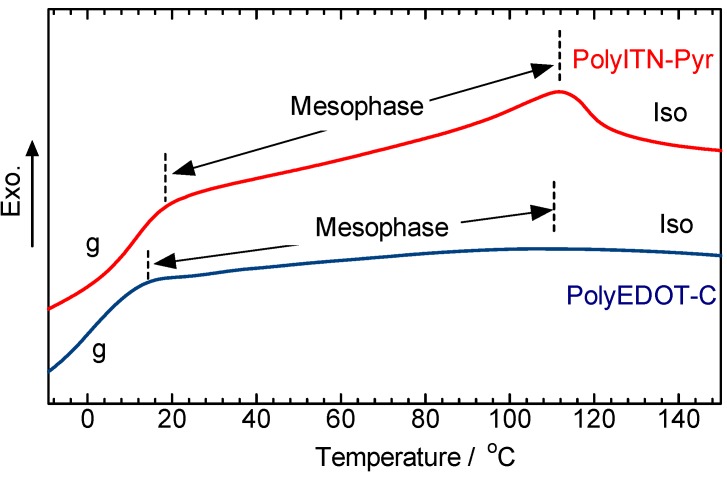
Differential scanning calorimetry (DSC) measurement results of polyITN-Pyr and polyEDOT-C in second cooling process at a scan rate of 10 °C/min. g = glassy state, mesophase = LC phase, Iso = isotropic.

Figure 7 shows differential scanning calorimetry (DSC) measurement results of polyITN-Pyr and polyEDOT-C in the second cooling process at a scan rate of 10 °C/min. The results indicate that polyITN-Pyr and polyEDOT-C can exhibit thermotropic liquid crystallinity in the cooling process. The polymers show no transition signal from mesophase to solid state. The polymers at room temperature may be in an LC state with quite high viscosity, or the polymers solid state, showing no clear transition signal (mesophase to solid) in the DSC. The optical textures of the polymers correspond to the mesophase. Further investigation on the DSC studies may be required. In the present stage, we could not synthesize the samples in large amounts for further investigation.

**Figure 8:**
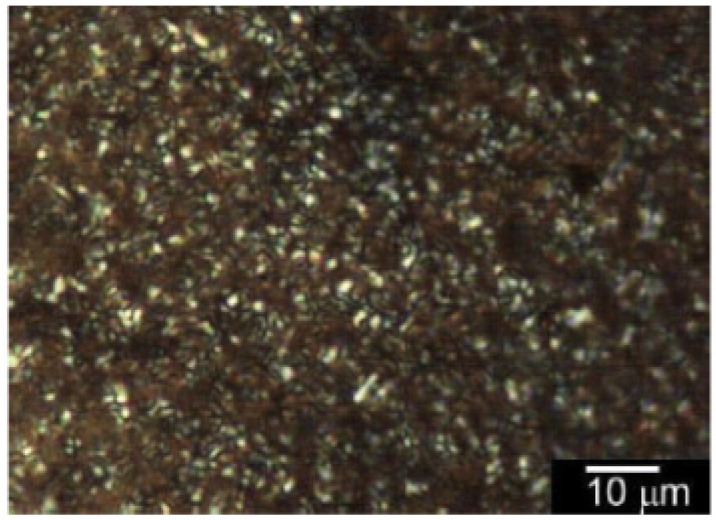
Polarizing optical microscopy (POM) images of polyITN-Pyr cast film from THF solution.

PolyEDOT-C shows no lyotropic LC (liquid crystallinity in solvents), while polyITN-Pyr exhibits lyotropic liquid crystallinity in THF. The optical LC texture remained after completion of the evaporation of solvents via formation of LC state. In other words, the cast film prepared by solution evaporation of polyITN-Pyr from the THF solution possesses LC order. Figure 8 displays POM images of polyITN-Pyr cast film from THF solution. Small pebble-like structure of the polyITN-Pyr exhibits birefringence under the POM.

## 4. Conclusions

Poly(isothianaphthene-*alt*-(phenylene having LC groups)) and poly(3,4-ethylenedioxythiophene- *alt*-(phenylene having LC groups)) were synthesized via Migita-Kosugi-Stille polycondensation reaction. The IR absorption, the UV-vis optical absorption, and the PL spectroscopy measurements were carried out, evaluating the optical properties of the polymers. We confirmed the thermotropic LC nature of the polymers with a bandgap of 2.51 eV (polyITN-Pyr) and 2.21 eV (polyEDOT-C).
